# Kinetics of Germination of Individual Spores of *Geobacillus stearothermophilus* as Measured by Raman Spectroscopy and Differential Interference Contrast Microscopy

**DOI:** 10.1371/journal.pone.0074987

**Published:** 2013-09-13

**Authors:** Tingting Zhou, Zhiyang Dong, Peter Setlow, Yong-qing Li

**Affiliations:** 1 Institute of Microbiology, Chinese Academy of Sciences, Beijing, China; 2 Department of Molecular, Microbial and Structural Biology, University of Connecticut Health Center, Farmington, Connecticut, United States of America; 3 Department of Physics, East Carolina University, Greenville, North Carolina, United States of America; Loyola University Medical Center, United States of America

## Abstract

*Geobacillus stearothermophilus* is a gram-positive, thermophilic bacterium, spores of which are very heat resistant. Raman spectroscopy and differential interference contrast microscopy were used to monitor the kinetics of germination of individual spores of *G. stearothermophilus* at different temperatures, and major conclusions from this work were as follows. 1) The CaDPA level of individual *G. stearothermophilus* spores was similar to that of *Bacillus* spores. However, the Raman spectra of protein amide bands suggested there are differences in protein structure in spores of *G. stearothermophilus* and *Bacillus* species. 2) During nutrient germination of *G. stearothermophilus* spores, CaDPA was released beginning after a lag time (*T*_lag_) between addition of nutrient germinants and initiation of CaDPA release. CaDPA release was complete at *T*_release_, and Δ*T*_release_ (*T*_release_ – *T*_lag_) was 1–2 min. 3) Activation by heat or sodium nitrite was essential for efficient nutrient germination of *G. stearothermophilus* spores, primarily by decreasing *T*_lag_ values. 4) Values of *T*_lag_ and *T*_release_ were heterogeneous among individual spores, but Δ*T*_release_ values were relatively constant. 5) Temperature had major effects on nutrient germination of *G. stearothermophilus* spores, as at temperatures below 65°C, average *T*_lag_ values increased significantly. 6) *G. stearothermophilus* spore germination with exogenous CaDPA or dodecylamine was fastest at 65°C, with longer T_lag_ values at lower temperatures. 7) Decoating of *G. stearothermophilus* spores slowed nutrient germination slightly and CaDPA germination significantly, but increased dodecylamine germination markedly. These results indicate that the dynamics and heterogeneity of the germination of individual *G. stearothermophilus* spores are generally similar to that of *Bacillus* species.

## Introduction

Many components of the spore germination machinery are conserved between spore forming members of the *Bacillales* [1]. *Bacillus subtilis* spore germination can be initiated by a variety of chemicals, including nutrients, cationic surfactants, and enzymes, as well as by hydrostatic pressure [2]. Nutrient germinants for spore germination generally include amino acids, purine derivatives, and sugars, and are species and strain specific. These nutrient germinants interact with germination receptors (GRs) located in the inner spore membrane [2], stimulating the release of the spore core’s large (∼10% of spore dry wt) depot of pyridine-2,6-dicarboxylic acid (dipicolinic acid [DPA]) and divalent cations, predominantly Ca^2+^, which are likely present as a 1∶1 chelate (CaDPA) [3]. CaDPA in the core is released and replaced by water in stage I of spore germination, and CaDPA release then triggers stage II of germination, a major event which is the hydrolysis of spores’ peptidoglycan cortex by cortex lytic enzymes (CLEs) [2], [4]. Concomitant with cortex hydrolysis, the core’s full rehydration ultimately leads to resumption of enzyme activity, and initiation of metabolism and macromolecular synthesis in the core, and thus spore outgrowth [2], [5].

*B. subtilis* spores contain three major GRs, termed GerA, GerB and GerK, each of which contains A, B and C subunits all of which are required for GR function [2]. These GRs are encoded by three tricistronic operons, each of which appears to encode a single GR [1], [2]. The GerD protein is also essential for proper GR function, and the proteins encoded by the *spoVA* operon are essential for DPA uptake in sporulation and probably CaDPA release during germination as well [1], [6], [7]. *Geobacillus stearothermophilus* is a Gram-positive spore-forming thermophile. Genomic analysis suggests that *G. stearothermophilus* has clear homologs of the *B. subtilis* GR genes as well as *gerD* and *spoVAB, C, D* genes, and genes encoding the cortex lytic enzymes CwlJ and SleB [1], [8].

*G. stearothermophilus* spores are the most wet heat-resistant among spores of aerobic spore-forming bacteria, and can spoil a variety of types of foodstuffs [9]–[12]. These spores are also commonly used as a biological indicator to evaluate the effectiveness of sterilization processes, in particular wet heat. However, the germination of spores of *G. stearothermophilus* species is much less well studied than that of spores of *Bacillus* species. Limited studies have shown that *G. stearothermophilus* spores germinate in response to low mol wt nutrient germinants including amino acids, purine and pyrimidine nucleosides, and sugars. However, the kinetics of the germination of individual *G. stearothermophilus* spores and the heterogeneity among individual spores in a population has not been studied.

In this study, we investigated the nutrient and non-nutrient germination of multiple individual intact and decoated *G. stearothermophilus* spores at various temperatures. We also measured the CaDPA level and Raman spectra of individual *G. stearothermophilus* spores and compared these with those of spores of several *Bacillus* species, as well as effects of different activation methods on kinetics of germination of individual *G. stearothermophilus* spores. This work has provided new information on the dynamics of and the heterogeneity in the germination of *G. stearothermophilus* spores.

## Materials and Methods

### Bacterial Species Used and Spore Preparation

Spores of *G. stearothermophilus* NGB101 were prepared and purified as described previously [13]. The *Bacillus* species used in this work were *Bacillus subtilis* PS533 [14] and *Bacillus cereus* T (originally obtained from H.O. Halvorson). Spores of these species were prepared and stored as described [15], [16]. All spores used in this work were free (>98%) of growing or sporulating cells, as determined by phase contrast microscopy.

### Measurement of CaDPA Level and Raman Spectra of Individual Spores by Laser Tweezers Raman Spectroscopy

The CaDPA levels of individual spores of various species were determined by laser tweezers Raman spectroscopy at 25°C [17]. Briefly, an individual spore was captured with laser tweezers, and its Raman spectrum was acquired with an integration of 20 s and a laser power of 20 mW at 780 nm. Spectra of 30 individual spores were measured and averaged. The CaDPA level in an individual spore was determined from the peak intensity at 1,017 cm^−1^ in its Raman spectrum relative to the peak intensity of the same Raman band from a CaDPA solution of known concentration (50 mM) and by multiplying this concentration value by the excitation volume of 1 fl to obtain attomoles of CaDPA/spore [17]. Raman spectra of 30 individual spores of *G. stearothermophilus*, *B. subtilis* and *B. cereus* at 25, 65, and 95°C were also averaged for analysis of heat-induced changes in spores’ molecular components.

### Activation of *G. stearothermophilus* Spores

Unless noted otherwise, prior to germination experiments, *G. stearothermophilus* spores were activated by one of three methods: 1) incubation in water at 100°C for 30 min followed by cooling in ice water for 15 min; 2) incubation in water at 30°C for 120 h; or 3) incubation in 0.2 M sodium nitrite (pH 8.0) at 30°C for 17 h. Germination of unactivated *G. stearothermophilus* spores was also carried out in a few experiments.

### Monitoring Germination of Single Spores by Raman Spectroscopy and Differential Interference Contrast (DIC) Microscopy

The germination of an individual *G. stearothermophilus* spore with 0.1 mM L-valine in 10 mM sodium phosphate buffer (pH 8.0) at 65°C was monitored simultaneously by Raman spectroscopy and DIC microscopy, as described [18], [19]. Briefly, a single *G. stearothermophilus* spore was optically captured immediately after the addition of 65°C 0.1 mM L-valine/10 mM sodium phosphate buffer. Both the Raman spectra and DIC microscopy images of the trapped spore were recorded simultaneously for a period of 45 min with intervals of 30 s per spectrum and 15 s per image frame, respectively. Note that the low concentration of L-valine used in this experiment was to slow spore germination sufficiently to allow its measurement by Raman spectroscopy.

### Monitoring Germination of Multiple Individual Spores by DIC Microscopy

The germination of a number of individual spores was simultaneously monitored with DIC microscopy [18]. Prior to germination, the spores were routinely activated at 100°C for 30 min unless noted otherwise. Briefly, 1 µl of heat-activated spores (10^8^ spores/ml in water) was spread on the surface of a glass coverslip glued to a clean and sterile sample container. The spores on the container were quickly dried in a vacuum chamber at room temperature so that they adhered to the coverslip. The spore container was then mounted on a microscope heat stage kept at the appropriate temperature. Preheated germinant / buffer solution was then added to the container, and a digital CCD camera (12 bits; 1600 by 1200 pixels) was used to record the DIC images at a rate of 1 frame per 15 s for 60–120 min. These DIC images were analyzed with a computation program in Matlab to locate each spore’s position and to calculate the summed pixel intensity. The DIC image intensity of each spore was plotted as a function of the incubation time (with a resolution of 15 s).

Unless noted otherwise, *G. stearothermophilus* spores were germinated at various temperatures in: (i) 1 mM L-valine in 10 mM sodium phosphate buffer (pH 8.0); (ii) 1 mM AGFK (a mixture of 1 mM each of L-asparagine, D-glucose, D-fructose, and potassium ions) in 10 mM sodium phosphate buffer (pH 8.0); (iii) 60 mM CaDPA made to pH 7.4 with Tris base; and (iv) 1 mM dodecylamine in 10 mM sodium phosphate buffer (pH 8.0). Except for dodecylamine and CaDPA germination, spores were routinely activated for 30 min at 100°C prior to germination experiments unless noted otherwise.

### Chemical Decoating of G. stearothermophilus Spores and Germination of Decoated Spores

Spores of *G. stearothermophilus* at an optical density at 600 nm of ∼10 were decoated by treatment with 1% sodium dodecylsulfate (SDS)–0.1 M NaOH–0.1 M NaCl–0.1 M dithiothreitol for 30 min at 65°C [20]. This procedure removes much of the spore’s coat protein as well as the spore’s outer membrane [20]. The decoated spores were washed at least 10 times with 0.1 M NaCl by centrifugation to remove all traces of the decoating solution and suspended in water. The decoated spores were then germinated with various agents with or without activation treatment as described above.

### Data Analysis

The DIC microscope that monitored individual spores was set such that the polarizer and analyzer were crossed, and thus the DIC bias phase was zero. After adding pre-heated germinant/buffer solution to spores on the coverslips, a digital CCD camera was used to record the DIC images. These images were analyzed with a Matlab program to locate each spore’s position and to calculate the averaged pixel intensity of an area of 20×20 pixels that covered the whole individual spore on the DIC image. The DIC image intensity of each individual spore was plotted as a function of the incubation time and the initial intensity (the first DIC image recorded after the addition of the germinant) was normalized to 1 and the intensity at the end of measurements was normalized to zero. Invariably, the latter value had been constant for ≥10 min at the end of measurements.

From the time-lapse DIC image intensity, we can determine the time of completion of the rapid fall of ∼75% in spore DIC image intensity, which is concomitant with the time of completion of spore CaDPA release (*T*_release_). CaDPA release kinetics during germinationof individual spores were described by the parameters *T*_lag_, *T*_release_ and Δ*T*_release_ [7], [18]. We also defined the additional germination parameters, *T*_lys_ and Δ*T*_lys_ where *T*_lys_ is the time when spore cortex hydrolysis is completed as determined by the completion of the fall in the spore’s DIC image intensity, and Δ*T*_lys_ = (*T*_lys_-*T*_release_).

## Results

### Raman Spectra and Average CaDPA Level of Individual G. Stearothermophilus Spores

**Figure 1:**
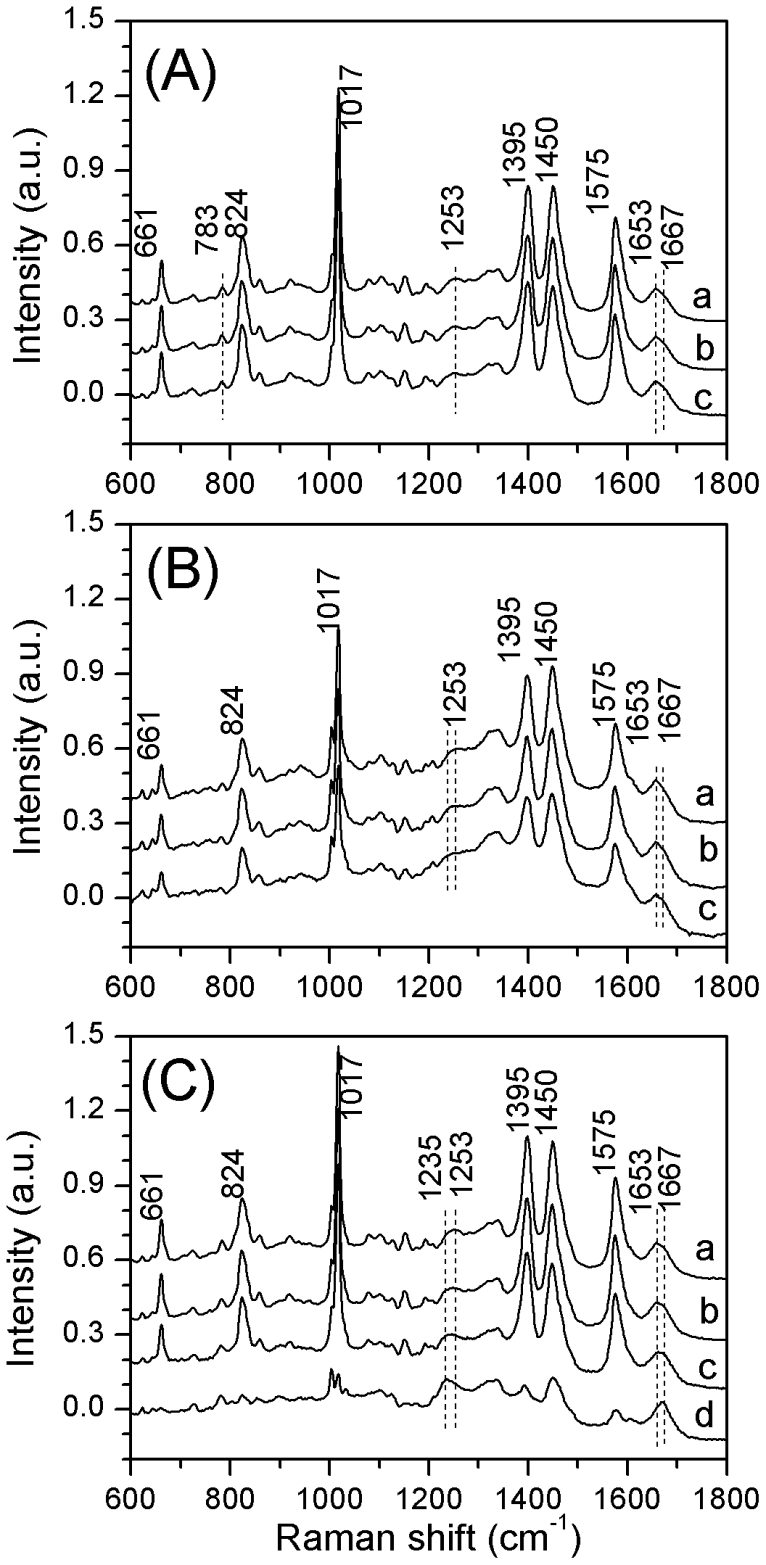
Raman spectra of individual spores. Raman spectra of individual *G. stearothermophilus* (A), *B. subtilis* (B), and *B. cereus* spores (C), measured at 25°C (curve a), 65°C (curve b) and 95°C (curve c), respectively. Curve d in Fig. 1(C) is the Raman spectrum of single *B. cereus* spores that had lost their CaDPA at 95°C. All the spectra were averages from 30 individual spores determined as described in Methods. The dotted lines are the protein bands of amide I (1653/1667 cm^−1^) and amide III (1253 cm^−1^), respectively.

CaDPA dominates the Raman spectra of individual spores of *Bacillus* species [21], and this was also the case for spores of *G. stearothermophilus* (Fig. 1). The intensity of the CaDPA-specific 1,017 cm^−1^ Raman band in the average spectrum from 30 individual spores indicated that the CaDPA level in the core of *G. stearothermophilus* spores was ∼ 382 mM, and this value was only slightly higher than the values for *B. subtilis* and *B. cereus* spores (Table 1).

**Table 1:** CaDPA level in individual *G. stearothermophilus* and *Bacillus* spores*.

Spores	CaDPA level (mM)
*G. stearothermophilus*	382±79
*B. cereus*	350±105
*B. subtilis*	335±42

* CaDPA levels in 30 individual spores of various *Bacillales* species were determined, and mean values and standard deviations were calculated as described in Materials and Methods.

Fig. 1 also shows the average Raman spectra of individual *G. stearothermophilus* spores measured at 25, 65, and 95°C, in comparison to spectra of *B. subtilis* and *B. cereus* spores. The peaks at 661, 824, 1,017, 1,395 and 1,575 cm^−1^ are the bands due to CaDPA, while the dotted lines denote the protein bands of amide I (1653/1667 cm^−1^) and amide III (1253 cm^−1^), respectively. The bands at 1,653 and 1,667 cm^−1^ are assigned to the α-helical and nonregular structures of the amide I (peptide bond C = O stretch) of proteins, respectively [22]–[24]. Raman spectra of *B. subtilis* and *B. cereus* spores (Fig. 1(B, C)) show that as the temperature was increased from 25 to 95°C, the intensity of the 1653 cm ^−1^ band was slightly decreased and the intensity at 1667 cm^−1^ slightly increased. Similarly, the peak of the 1253 cm^−1^ band (protein amide III) was slightly shifted to the left at the higher temperatures. This suggests that the structure of proteins in *B. subtilis* and *B. cereus* spores had changed significantly from an α-helical structure to a nonregular structure at high temperature, indicative of significant denaturation of proteins in these spores as found previously [24]–[26]. Indeed, when incubated at 95°C, some *B. cereus* spores lost their CaDPA, the 1653c m^−1^ band shifted to 1667 cm^−1^, and the 1253 cm^−1^ band shifted to 1235 cm^−1^ (curve d in Fig. 1(C)), suggesting that significant protein denaturation took place after CaDPA release at 95°C. In contrast to results with *B. subtilis* and *B. cereus* spores, the Raman bands of protein amide I (1653/1667 cm^−1^) were unchanged for *G. stearothermophilus* spores at 95°C (Fig. 1(A)), indicating that these spores’ proteins are stable even at 95°C, consistent with these spores’ extremely high wet heat resistance. The amide III band (1230–1300 cm^−1^) region centered at 1253 cm^−1^ shifted to a lower wavenumber at 65°C and at 95°C for *B. cereus* spores, but for *G. stearothermophilus* and *B. subtilis* spores, this change was less prominent. The Raman band at 783 cm^−1^ seen at 25°C is attributed to ring breathing of cytosine/thymine/uracil and the O–P–O symmetric stretch of the phosphodiester bond in DNA and RNA [27], [28]. At 95°C, the Raman band at 783 cm^−1^ was nearly unchanged, suggesting that the double helical structure of nucleic acids in *G. stearothermophilus* spores is stable at elevated temperature.

### Dynamics of Germination of Single *G. stearothermophilus* Spores

**Figure 2:**
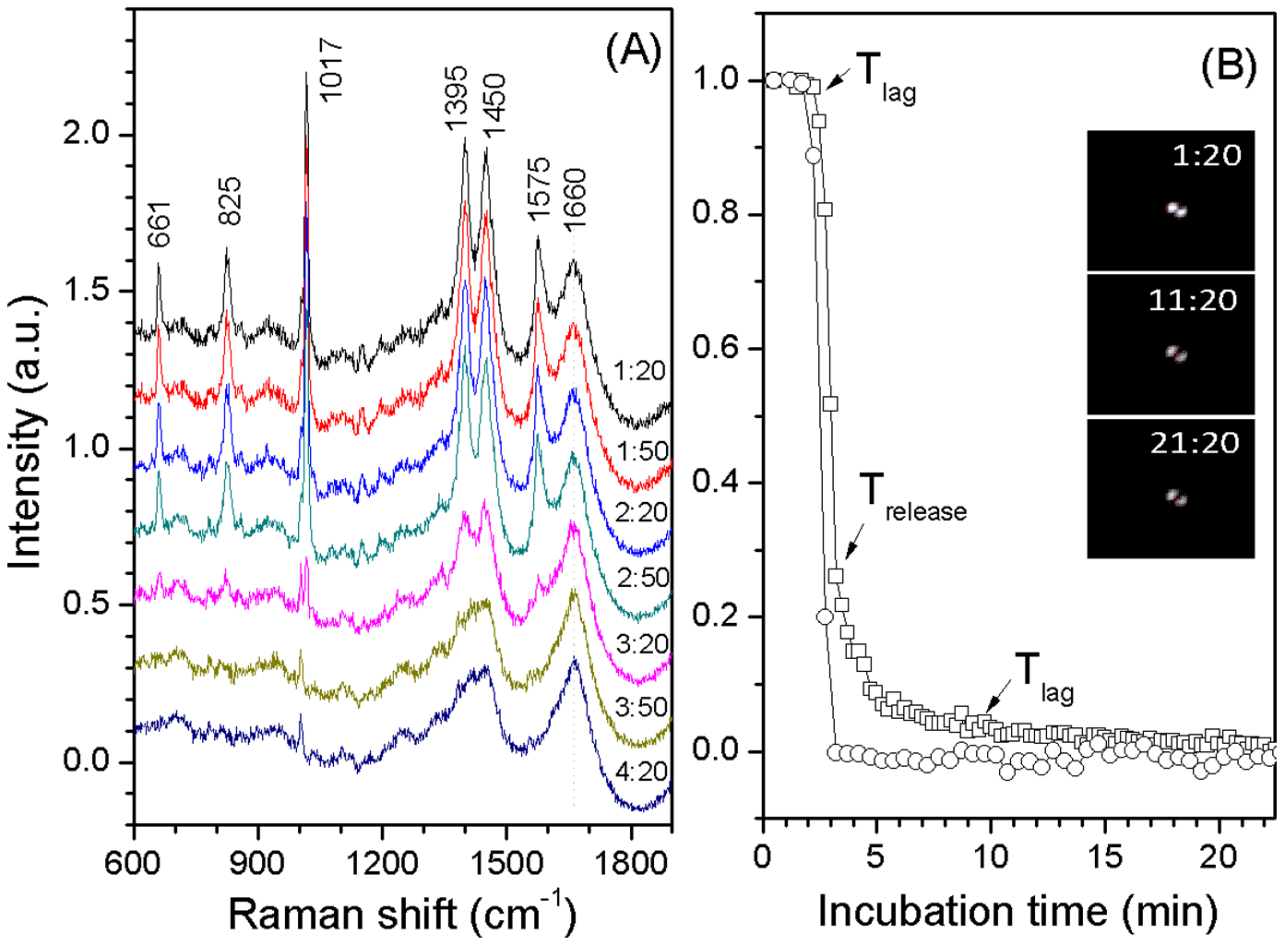
Dynamics of nutrient germination of an optically trapped individual *G. stearothermophilus* spore. A heat activated (30 min, 100°C) spore was germinated at 65°C with 0.1 mM L-valine in 10 mM sodium phosphate buffer (pH 8.0), and the spore was monitored by Raman spectroscopy and DIC microscopy as described in Methods. Time-lapse Raman spectra of the trapped spores after the addition of L-valine were shown in (A). The indicated peaks at 661, 825, 1,017, 1,395 and 1,575 cm^−1^ are the CaDPA bands. Normalized intensities of the CaDPA band at 1017^−1^ (○) and DIC images (□) as the function of incubation time were shown in (B). The CaDPA band intensities and DIC image intensities were normalized to their initial values right after the addition of L-valine, and the DIC image intensity at 25 min was normalized to 0. The interval between Raman spectrum acquisitions was 30 s, and the interval between DIC image acquisitions was 15 s. The inserts in Fig. 2(B) are the time-lapse DIC images of the trapped spore with a scale bar of 2 µm. The DIC image of a single spore appears as two bright spots in DIC microscopy.

Fig. 2 shows dynamics of an optically trapped individual *G. stearothermophilus* spore during L-valine germination at 65°C, as monitored by Raman spectroscopy and DIC microscopy. After the addition of the germinant the CaDPA level as measured by the 1017 cm^−1^ band [17] and the DIC image intensity were nearly unchanged before *T*_lag_ at ∼ 2.2 min. The intensity of the 1017 cm^−1^ band then quickly dropped to zero and the spore’s DIC image intensity decreased ∼70% by *T*_release_ at ∼ 3.2 min. In this experiment, the DIC image intensity of the *G. stearothermophilus* spore usually continued to fall (but see below) until T_lys_ at ∼ 9.6 min, corresponding to the completion of spore cortex hydrolysis, and then remained constant. As seen with the germination of *Bacillus* spores [19], the termination point of the rapid fall in DIC image intensity precisely corresponded to the completion of CaDPA release for *G. stearothermophilus* spores.

### Effect of Different Activation Methods on *G. stearothermophilus* Spore Germination

**Figure 3:**
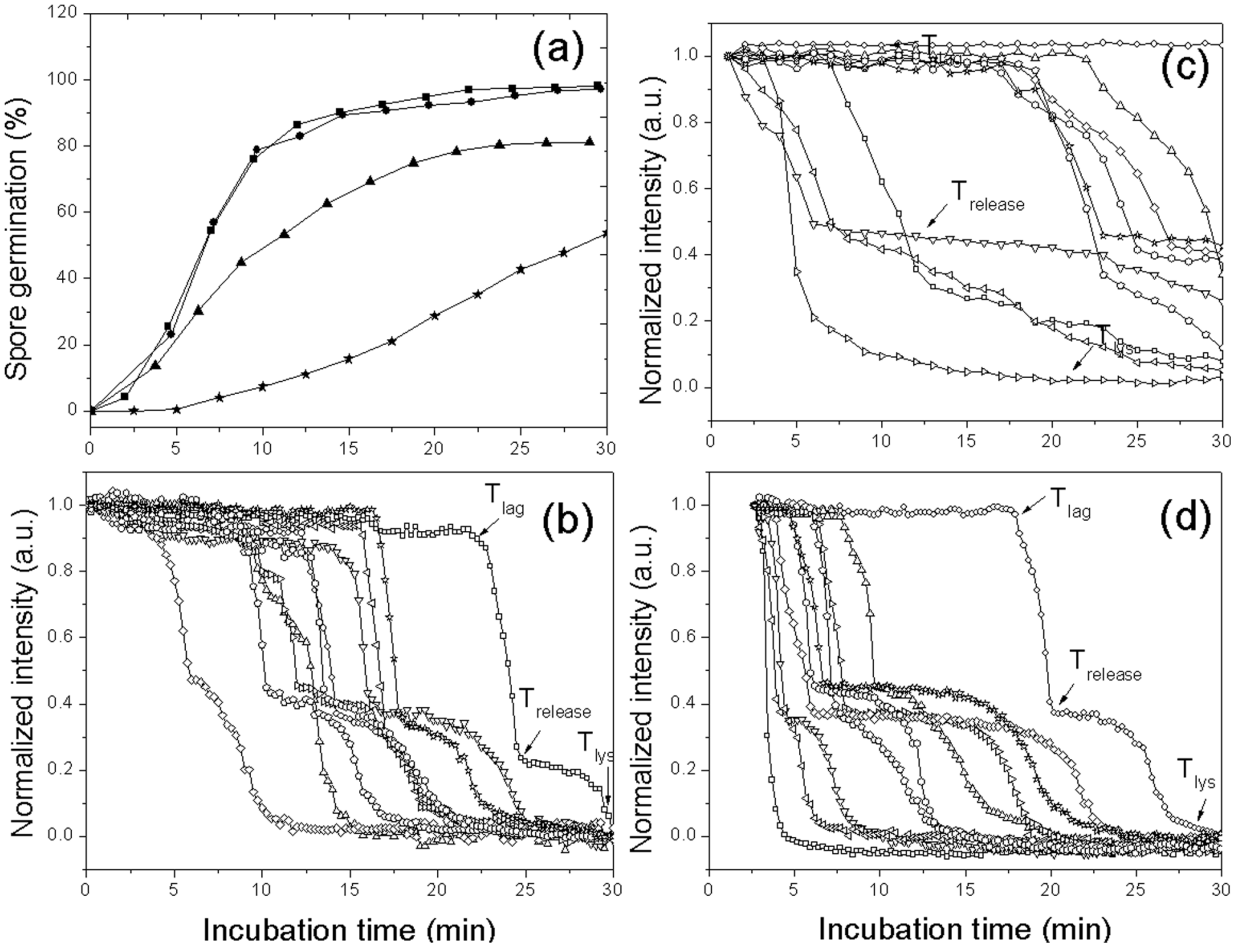
Effects of different activation methods on germination of multiple individual *G. stearothermophilus* spores. Spores were activated by various methods, and germinated at 65°C with 1.0 mM L-valine and 10 mM sodium phosphate buffer (pH 8.0), and germination of individual spores was monitored by DIC microscopy as described in Methods. Germination of ≥248 individual spores (Table 2) that were activated in 0.2 M sodium nitrite (pH 8.0) at 30°C for 17 h (•), in water at 30°C for 120 h (▴), in water at 100°C for 30 min (▪), or without activation (*) was shown in (a). Kinetics of germination of ten individual spores without activation (b); activated at 30°C for 120 h (c), and activated in 0.2 M sodium nitrite (pH 8.0) for 17 h (d) was given in (b-d).

Previous studies [29], [30] have shown that germination of *G. stearothermophilus* spores becomes much more rapid if the spores are first given an activation treatment such as incubation in water for short times at a high temperature, long times in water at a moderate temperature, or incubation in sodium nitrite at a moderate temperature for intermediate times. The current work demonstrated that these different activation regimens led to different kinetics of L-valine germination of individual *G. stearothermophilus* spores at 65°C (Table 2; Fig. 3). All three activation regimens increased the overall rates of spore germination, almost completely by decreasing average *T*_lag_ values with minimal if any effects on values for Δ*T*_release_ and Δ*T*_lys_. Note also that for a number of the individual spores activated by various regimens, following the initial rapid fall in DIC image intensity of ∼ 60%, there was an lag of 5–20 min following *T*_release_ and before the further fall in DIC image intensity. This was also seen in many other germination experiments (see below), although the reason for this lag period is not clear.

**Table 2:** Effect of activation methods on *G. stearothermophilus* spore germination*.

Activation method	No. of spores examined(% spore germination)	*T*_lag_ (min)	*T*_release_(min)	Δ*T*_release_(min)	*T*_lys_	Δ*T*_lys_(min)
No activation	458 (53.7)	12.6±6.2	14.1±6.2	1.4±0.8	19.5±6.2	5.4±2.8
100°C, 30 min	264 (98.1)	5.0±3.9	6.4±4.0	1.4±0.8	13.1±5.6	6.7±3.4
30°C, 0.2 M NaNO_2_, 17 h	248 (97.2)	4.4±3.2	5.5±3.3	1.1±0.6	11.5±5.6	6.0±2.9
30°C, 5 d	523 (81.1)	6.2±4.3	7.4±4.3	1.2±0.6	13.6±5.8	6.2±3.5

* Activated or unactivated spores were germinated at 65°C with 1 mM L-valine in 10 mM sodium phosphate buffer (pH 8.0) for 30 min, and kinetic parameters for all germinations were determined by analysis of ≥248 spores that germinated as described in Methods.

### Kinetics of Germination of Multiple Individual *G. stearothermophilus* Spores with L-valine or AGFK

**Figure 4:**
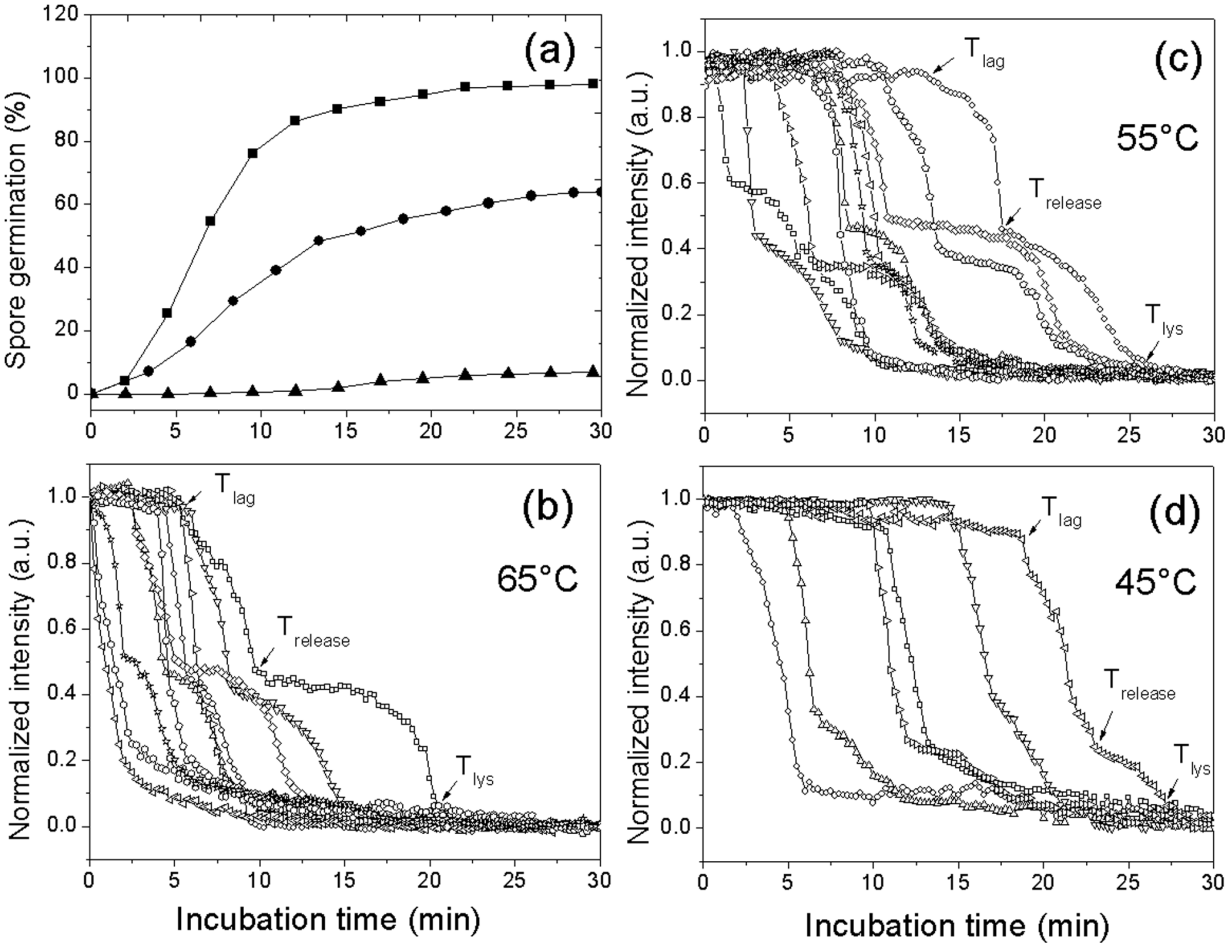
L-Valine germination of multiple individual *G. stearothermophilus* spores. Heat activated spores (30 min, 100°C) were germinated at various temperatures with 1 mM L-valine in 10 mM sodium phosphate buffer (pH 8.0), and germination of individual spores was monitored by DIC microscopy as described in Methods. Germination of ≥264 individual spores at 65°C(▪), 55°C (•), or 45°C (▴) was shown in (a). Kinetics of germination of ten individual spores at 65°C (b), 55°C (c), or 45°C (d) was given in (b–d).

**Figure 5:**
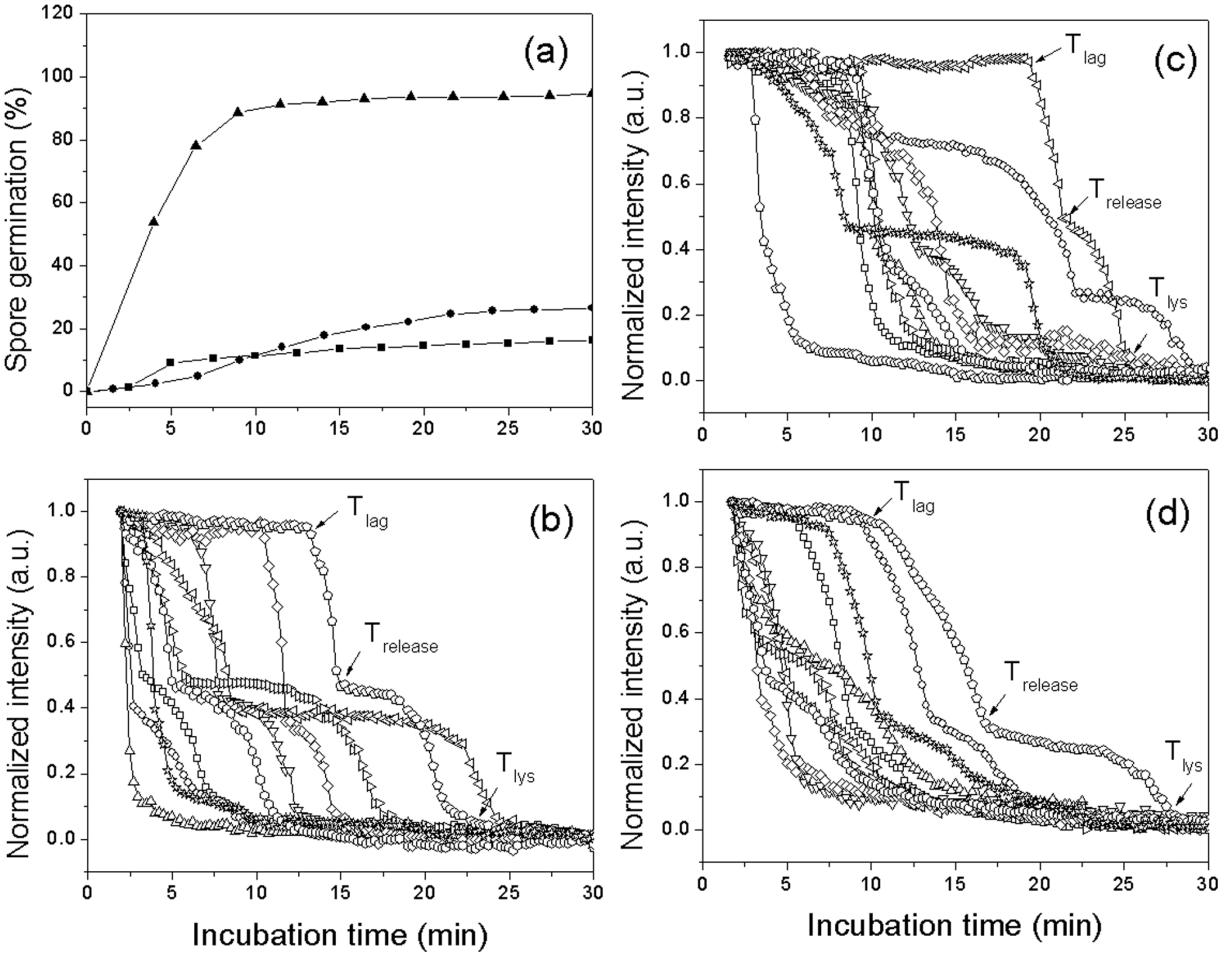
Germination of multiple individual *G. stearothermophilus* spores with AGFK at different temperatures. Heat activated (30 min, 100°C) spores were germinated at various temperatures with AGFK and 10 mM sodium phosphate buffer (pH 8.0), and germination of individual spores was monitored by DIC microscopy as described in Methods. Germination of ≥220 individual spores at 65°C (▴), 55°C (•), or 45°C (▪) was shown in (a). Kinetics of germination of ten individual spores at 65°C (b), 55°C (c), or 45°C (d) was given in (b–d).

Previous work [29] has shown that *G. stearothermophilus* spores are able to germinate in the presence of L-valine or AGFK. Consequently we used DIC microscopy to analyze the germination of multiple individual *G. stearothermophilus* spores with L-valine or AGFK at multiple temperatures (Fig. 4 and 5; Table 3; and data not shown). *G. stearothermophilus* spores germinated faster with AGFK than L-valine at 65°C, and as expected, germination with these nutrients was faster at 65°C than at 55°C or 45°C, while no germination was observed when *G. stearothermophilus* spores were incubated with 1 mM L-valine at 37°C or 25°C (Table 3; and data not shown). The slower germination of these spores at lower temperatures was due primarily to longer *T*_lag_ values as: i) many spores did not even germinate in the observation times at the lower temperatures, and thus have very long *T*_lag_ values; ii) Δ*T*_release_ values increased only slightly at lower temperatures; and iii) Δ*T*_lys_ values were essentially unchanged at low and high temperatures.

**Table 3:** Mean values and standard deviations of *T*_lag_, *T*_release_, Δ*T*_release_, *T*_lys, and_ Δ*T*_lys_ values for individual germinating *G. stearothermophilus* spores*.

Strains and germination conditions		No. of spores examined (% spore germination)	*T*_lag_ (min)	*T*_release_ (min)	Δ*T*_release_ (min)	*T*_lys_	Δ*T*_lys_ (min)
1 mM L-valine	65°C, 30 min	264 (98.1)	5.0±3.9	6.4±4.0	1.4±0.8	13.1±5.6	6.7±3.4
	55°C, 30 min	351 (63.8)	9.5±6.6	11.2±6.6	1.7±0.8	16.7±6.7	5.4±3.1
	45°C, 30 min	275 (6.9)	10.6±6.7	12.8±6.1	2.2±1.0	16.8±6.9	4.0±1.8
	Decoated, 65°C, 30 min	224 (89.7)	8.3±5.0	10.1±5.6	2.8±1.9	20.8±6.5	9.7±6.8
1 mM AGFK	65°C, 30 min	302 (94.7)	3.0±1.7	4.3±2.0	1.3±0.8	10.6±5.2	6.3±4.3
	55°C, 30 min	407 (26.5)	6.8±4.5	8.5±4.6	1.7±0.6	13.8±5.1	5.3±2.0
	45°C, 30 min	220 (16.4)	7.8±8.2	11.3±9.0	3.6±2.5	17.4±7.6	6.1±10.7
	Decoated, 65°C, 30 min	396 (76.0)	5.5±5.0	7.9±5.4	2.3±1.4	20.3±7.9	12.4±6.8
60 mM CaDPA (no activation)	65°C, 120 min	380 (99.0)	4.4±3.9	5.3±3.8	2.5±1.2	7.8±3.7	0.9±0.5
	25°C, 120 min	510 (68.8)	56.9±26.5	60.4±26.8	3.5±1.1	77.2±26.4	23.4±9.1
	Decoated, 65°C, 120 min	310 (66.1)	29.4±25.8	31.2±25.9	1.8±1.7	44.2±29.8	13.0±12.0
1 mM Dodecylamine(no activation)	65°C, 120 min	557 (54.0)	21.7±17.7	23.4±17.9	1.7±1.1	36.3±22.9	12.9±13.1
	55°C, 120 min	470 (11.5)	14.9±19.8	16.7±20.0	1.9±1.3	39.8±23.0	23.0±14.5
	45°C, 120 min	515 (3.9)	67.8±30.1	68.7±30.2	0.9±0.8	78.5±32.1	9.9±5.4
	Decoated, 65°C, 30 min	432 (94.9)	5.1±3.1	8.3±3.4	3.2±1.5	–	–

* Heat-activated *G. stearothermophilus* spores were germinated for 30 min with 1 mM L-valine or 1 mM AGFK at 65°C in 10 mM sodium phosphate buffer (pH 8.0), unactivated *G. stearothermophilus* spores were germinated at 65°C for 120 min with 60 mM CaDPA or with 1 mM dodecylamine in 10 mM sodium phosphate buffer (pH 8.0), and decoated *G. stearothermophilus* spores (heat-activated or unactivated) were germinated with different germinants at 65°C for 30 or 120 min. Kinetic parameters for individual germinations were determined by analysis of ≥100 spores that germinated as described in Methods.

### Kinetics of Non-nutrient Germination of Individual *G. stearothermophilus* Spores

**Figure 6:**
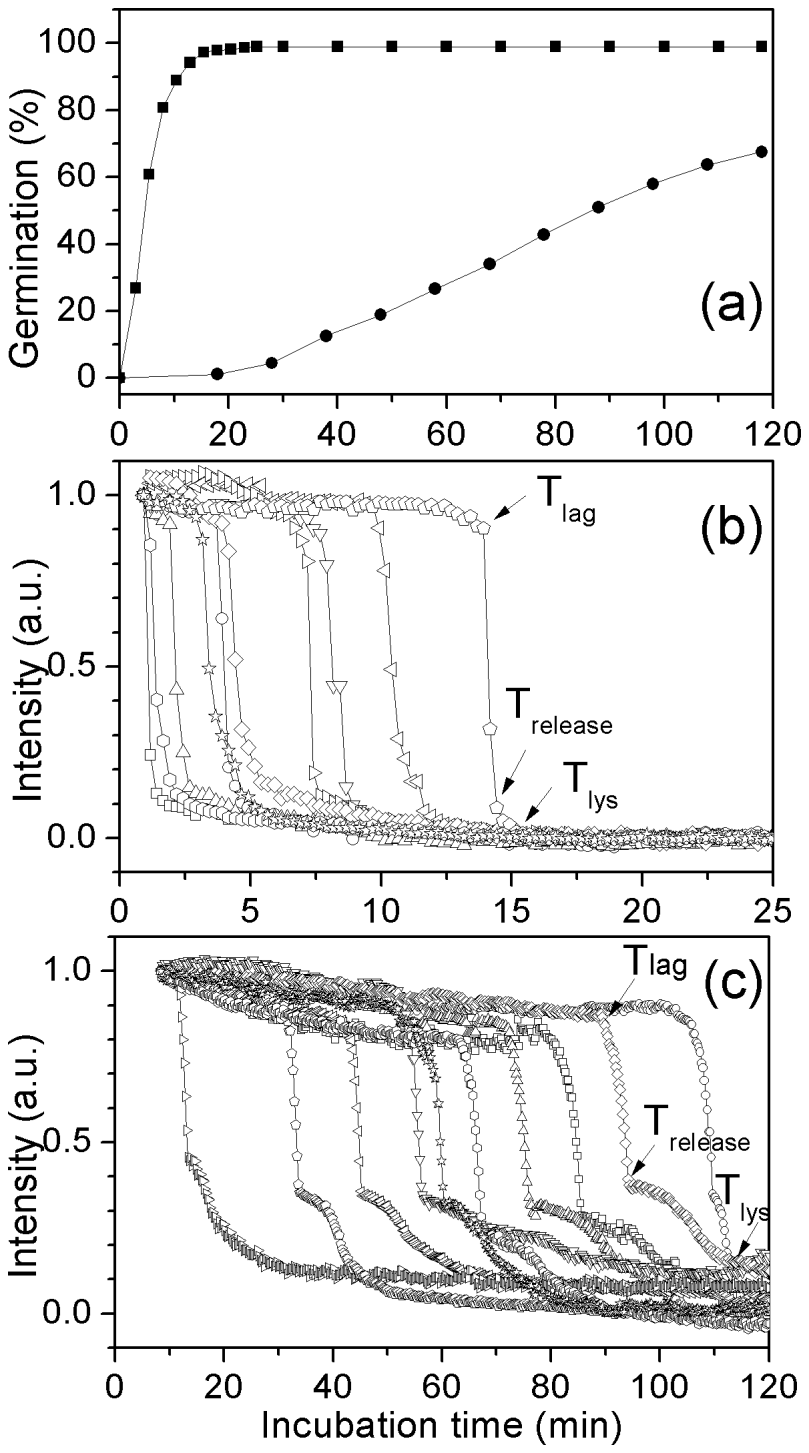
Germination of multiple individual *G. stearothermophilus* spores with CaDPA. Unactivated spores were germinated with CaDPA at various temperatures, and germination of individual spores was followed by DIC microscopy as described in Methods. Germination at 65°C (▪) or 25°C (•), with germination of ≥380 individual spores examined was shown in (a). Kinetics of germination of ten individual spores at 65°C (b) or25°C (c) was given in (b, c).

In addition to nutrients, spores can germinate with a variety of non-nutrients [2], [3], including lysozyme, CaDPA, cationic surfactants, high pressures and some salts. Unlike the case with nutrient germination, exogenous CaDPA induced germination of *G. stearothermophilus* spores at 25°C (Fig. 6; Table 3). However, CaDPA germination of *G. stearothermophilus* spores was faster at 65°C due largely to a much shorter average *T*_lag_ value than at 25°C, although the Δ*T*_release_ values were almost identical at these two temperatures. The average Δ*T*_lys_ value for CaDPA germination at 25°C was also much longer than for CaDPA germination at 65°C.

**Figure 7:**
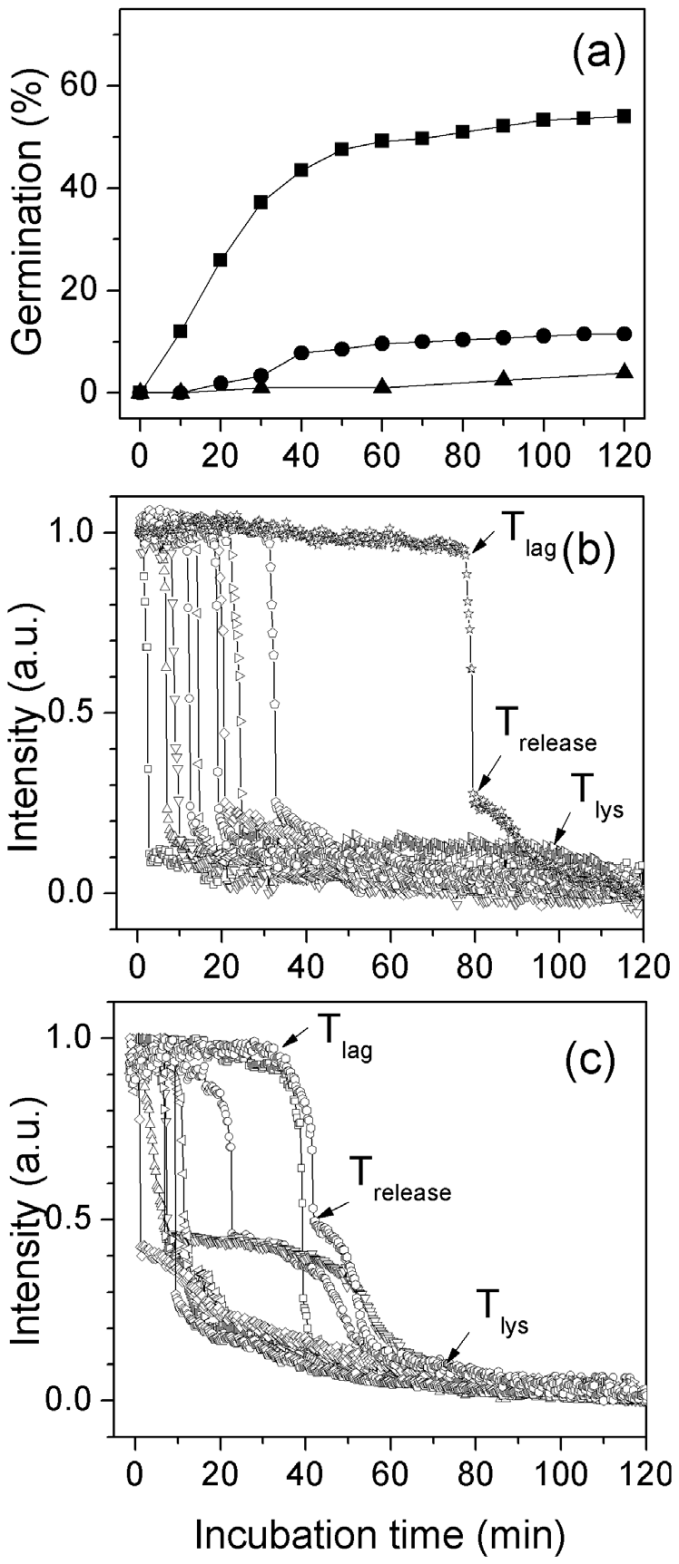
Germination of multiple individual *G. stearothermophilus* spores with dodecylamine. Unactivated spores were germinated at various temperatures with 1(pH 8.0), and germination of ≥470 individual spores was monitored by DIC microscopy. Germination at 65°C (▪), 55°C (•) and 45°C (▴) was shown in (a). Kinetics of germination of ten individual spores at 65°C (b) or 55°C (c) was given in (b, c).

Another group of non-nutrient germinants is cationic surfactants, with dodecylamine being the one that has been best studied [31].With 1 mM dodecylamine at 65°C, only ∼ 50% of *G. stearothermophilus* spores germinated in 120 min, a slow germination compared to those with other germinants, and dodecylamine germination was minimal at 45°C (Fig. 7; Table 3). As seen with CaDPA germination at low and high temperatures, most of the decrease in the rate of germination with dodecylamine at the lower temperature was due to much longer average *T*_lag_ values.

### Kinetics of Germination of Individual Decoated *G. stearothermophilus* Spores

**Figure 8:**
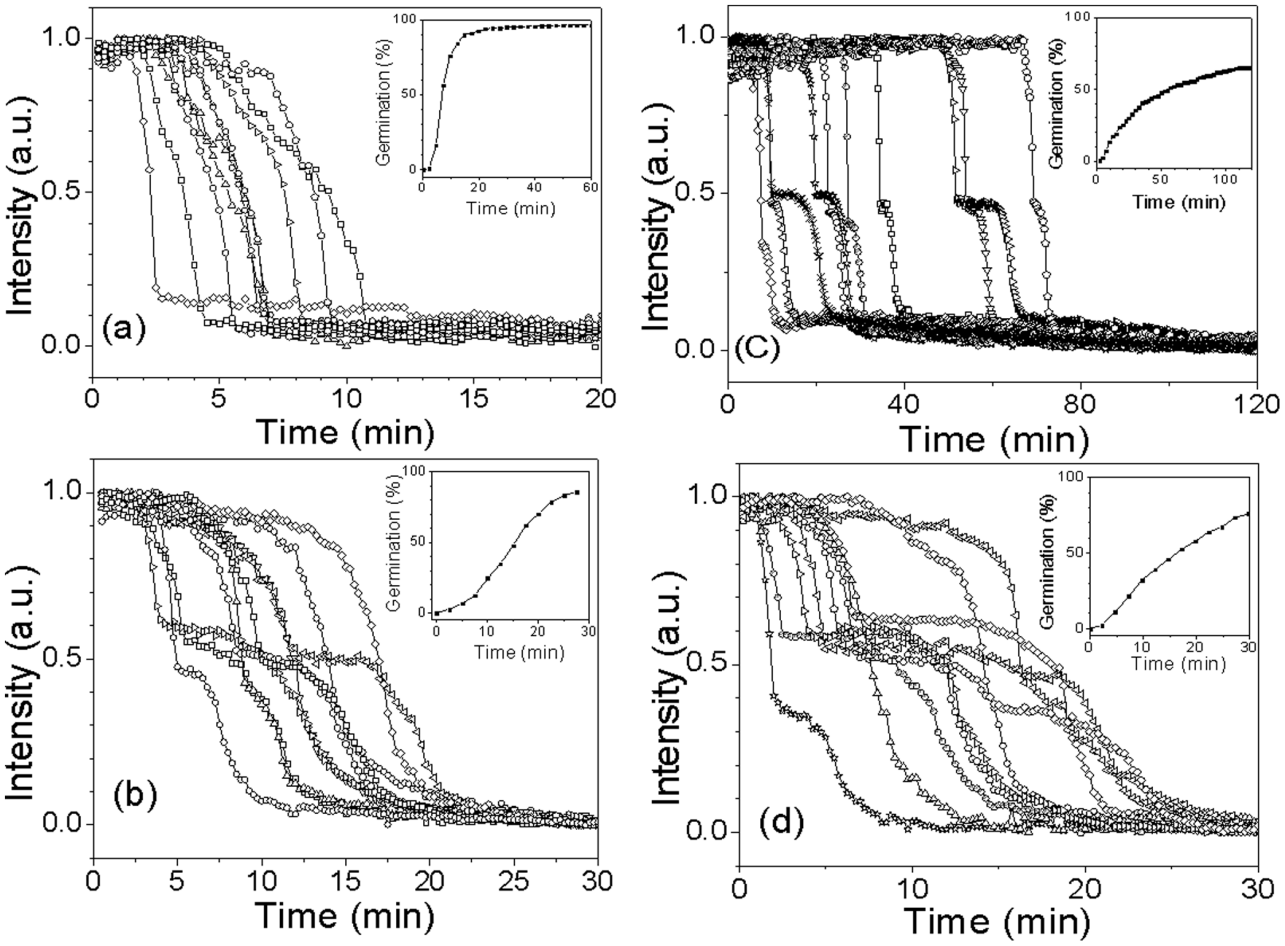
Germination of multiple individual decoated *G. stearothermophilus* spores. Heat activated (30 min, 100°C (a,b), or unactivated spores (c,d) were germinated at 65°C with 1 mM L-valine (a); 1 mM AGFK (b); 60 mM CaDPA (c); and 1 mM dodecylamine (d), in 10 mM sodium phosphate buffer (pH 8.0), and germination of individual spores was monitored by DIC microscopy as described in Methods. The insets in the various panels show the percentages of spore germination when ≥224 individual spores (Table 2) were monitored.

Since at least some proteins involved in spore germination in *Bacillus* species are located in the spore coats, in particular the CLE CwlJ [2], we also examined the effect of chemical decoating on *G. stearothermophilus* spores’ germination with nutrient and non-nutrient germinants, all at 65°C (Fig. 8; Table 3). With L-valine and AGFK, the rate of germination of decoated *G. stearothermophilus* spores decreased by ∼ 15%, while *T*_lag_ values increased ∼1.5 fold. However, the amount and rate of CaDPA germination of the decoated spores were markedly lower than with intact spores, as the average *T*_lag_ value increased >6-fold while the average *T*_lys_ value increased∼13-fold, although the average Δ*T*_release_ value was essentially unchanged from that for intact spores. Decoating also greatly increased the rate of dodecylamine germination of *G. stearothermophilus* spores markedly, largely by decreasing the average *T*_lag_ value (Table 3).

## Discussion

The work in this communication has revealed a number of similarities in the properties of spores of *G. stearothemophilus* and *Bacillus* species, in particular the nearly identical DPA concentrations in these spores’ core. However, there were some differences. One was the lack of change in the Raman spectrum of proteins in *G. stearothermophilus* spores upon incubation at 95°C. This behavior, as well as the high temperature needed for heat activation of *G. stearothermophilus* spores, is undoubtedly a reflection of *G. stearothermophilus* being a thermophile, and is consistent with both the high temperature optimum for germination of spores of this species and their extremely high wet heat resistance compared to spores of *B. cereus* and *B. subtilis* [10]. The second difference, and a more intriguing one was the highly variable lag period between *T*_release_ and the initiation of the second fall in *G. stearothermophilus* spores’ DIC image intensity during spore germination with all germinants tested, as this has been seen only rarely in germination of spores of *Bacillus* species [19], [32]. While we have no good explanation for this difference, it is as if there is a much higher threshold for the signal event that begins *G. stearothermophilus* spore cortex degradation by CLEs following CaDPA release than with spores of *Bacillus* species. However, these signaling mechanisms are not well understood, so we have no good mechanistic explanation for this difference between spores of these two genera.

While there were the notable differences between *G. stearothermophilus* and *Bacillus* spore properties noted above, the overall features of the nutrient and non-nutrient germination kinetics of individual spores of this species were very much like those of *Bacillus* species. Thus CaDPA release for all *G. stearothermophilus* spore germinations examined began only after a highly variable *T*_lag_ period but Δ*T*_release_ took only a few min, with *T*_release_ followed by cortex hydrolysis that was completed at *T*_lys_. Almost always, Δ*T*_lys_ was longer than Δ*T*_release_, and most of the heterogeneity in the germination between individual spores was in Δ*T*_lag_ values, as seen previously with spores of *Bacillus* species [18], [32], [33]. The effects of activation treatments on the germination *G. stearothermophilus* were also largely, if not completely on *T*_lag_ values, as average Δ*T*_release_ and Δ*T*_lys_ values in nutrient germination of unactivated and maximally activated spores were essentially identical. Optimal heat activation also decreases average *T*_lag_ values for nutrient germination of spores of *Bacillus* species [34]. Since a major factor determining the *T*_lag_ period for nutrient germination of spores of *Bacillus* species is spores’ levels of functional GRs [33], this further suggests that heat activation of *G. stearothermophilus* spores for 30 min at 100°C makes these spores’ GRs optimally functional, perhaps by some conformational protein changes as has been suggested for spores of *Bacillus* species [35]. The mechanism of nitrite activation of spores has never been analyzed in detail, but could be due to covalent modification of the spore cortex by nitrous acid [36]. However, this could equally well be due to nitrous acid modification of GRs.

It was also notable that germination at suboptimal temperatures greatly increased *T*_lag_ values for nutrient germination of *G. stearothermophilus* spores, especially given that lower percentages of these spores germinated at lower temperatures in the observation periods used. In contrast, there was essentially no effect on Δ*T*_lys_ values as the germination temperature was lowered, indicating that the temperature sensitive step in nutrient germination of *G. stearothermophilus* spores is in *T*_lag_, and probably is on the GRs themselves, although there was also a small increase in Δ*T*_release_ times as germination temperature was lowered. The effect of temperature on kinetics of the germination of individual spores has not been studied with spores of *Bacillus* species.

Decoating of *G. stearothermophilus* spores had only a minimal effect on their nutrient germination, with the biggest effect being 1.5 to 2-fold increases in Δ*T*_lys_ values. The *G. stearothermophilus* genome has the genes for the two redundant CLEs, CwlJ and SleB, involved in cortex hydrolysis during spore germination in *Bacillus* species. With *Bacillus* spores, decoating largely removes or inactivates CwlJ [37], and presumably a decrease in CwlJ level is the reason for the increased Δ*T*_lys_ values in decoated *G. stearothermophilus* spores. However, we do not know if all *G. stearothermophilus* CwlJ is inactivated by the decoating regimen we used. Indeed, decoating or loss of CwlJ by mutation increases values of Δ*T*_release_ in nutrient germination of spores of several *Bacillus* species 6- to 10-fold [38], [39], while the increase in decoated *G. stearothemophilus* spores was at most 2-fold. Thus with *G. stearothermophilus* spores either CwlJ is not essential for rapid CaDPA release in spore germination, or some active CwlJ survives the decoating regimen used. We favor the latter possibility, since CwlJ is essential for CaDPA germination of spores of *Bacillus* species [37], [40], while significant CaDPA germination still took place with decoated *G. stearothermophilus* spores. However, the average *T*_lag_ value for CaDPA germination increased ∼ 7-fold in decoated *G. stearothermophilus* spores. Thus it seems most likely that CwlJ is also the primary target of CaDPA in triggering germination of *G. stearothermophilus* spores.

Along with nutrient germination, *G. stearothermophilus* spore germination with the non-nutrients CaDPA and dodecylamine also decreased markedly at suboptimal temperatures, again largely due to effects on *T*_lag_. However, the latter effect is almost certainly not on GRs, which are not involved in CaDPA and dodecylamine germination of spores of *Bacillus* species [31], [37]. Indeed, as noted above, CaDPA probably triggers *G. stearothermophilus* spore germination by activating the CLE CwlJ, while in *Bacillus* spores dodecylamine likely triggers germination by triggering the opening of the CaDPA channel in the spores’ inner membrane that is composed at least in part of SpoVA proteins [41]. Interestingly, decoating of *G. stearothermophilus* spores significantly increased these spores’ germination with dodecylamine primarily by decreasing *T*_lag_ values, just as with spores of *Bacillus* species [31]. Why this should be is not completely clear, but decoating may allow easier access of dodecylamine to the SpoVA CaDPA channel than in an intact spore.

In summary, the analysis of the dynamics of the germination of multiple individual *G. stearothermophilus* spores with a variety of germinants indicates that the general features of the germination of these spores appear to be quite similar to those of spores of *Bacillus* species.

## References

[1] Paredes-Sabja D, Setlow P, Sarker MR (2011) Germination of spores of *Bacillales* and *Clostridiales* species: mechanisms and proteins involved. Trends Microbiol 19: 85–94. 21112786 10.1016/j.tim.2010.10.004

[2] Setlow P (2003) Spore germination. Curr Opin Microbiol 6: 550–556. 14662349 10.1016/j.mib.2003.10.001

[3] Paidhungat M, Setlow P (2002) Spore germination and outgrowth. In: Hoch JA, Losick R, Sonenshein AL, editors. *Bacillus subtilis* and its relatives: from genes to cells. Washington, DC: American Society for Microbiology. 537–548.

[4] Moir A (2006) How do spores germinate? J Appl Microbiol 101: 526–530. 16907803 10.1111/j.1365-2672.2006.02885.x

[5] Cowan AE, Koppel DE, Setlow B, Setlow P (2003) A soluble protein is immobile in dormant spores of *Bacillus subtilis* but is mobile in germinated spores: Implications for spore dormancy. Proc Natl Acad Sci USA 100: 4209–4214. 12646705 10.1073/pnas.0636762100 PMC404470

[6] Pelczar PL, Igarashi T, Setlow B, Setlow P (2007) Role of GerD in germination of *Bacillus subtilis* spores. J Bacteriol 189: 1090–1098. 17122337 10.1128/JB.01606-06 PMC1797312

[7] Wang G, Yi X, Li YQ, Setlow P (2011) Germination of individual *Bacillus subtilis* spores with alterations in the GerD and SpoVA proteins, which are important in spore germination. J Bacteriol 193: 2301–2311. 21398556 10.1128/JB.00122-11 PMC3133087

[8] Onyenwoke RU, Brill JA, Farahi K, Wiegel J (2004) Sporulation genes in members of the low G+C Gram-type-positive phylogenetic branch (Firmicutes). Arch Microbiol 182: 182–192. 15340788 10.1007/s00203-004-0696-y

[9] Feeherry F, Munsey DT, Rowley DB (1987) Thermal inactivation and injury of *Bacillus stearothermophilus* spores. Appl Environ Microbiol 53: 365–370. 3566270 10.1128/aem.53.2.365-370.1987 PMC203666

[10] Gerhardt P, Marquis RE (1989) Spore thermoresistance mechanisms. In: Smith I, Slepecky RA, Setlow P, editors. Regulation of prokaryotic development. Washington, DC: American Society for Microbiology. 43–63.

[11] Burgess SA, Lindsay D, Flint SH (2010) Thermophilic bacilli and their importance in dairy processing. Int J Food Microbiol 144: 215–225. 21047695 10.1016/j.ijfoodmicro.2010.09.027

[12] Prevost S, Andre S, Remize F (2010) PCR detection of thermophilic spore-forming bacteria involved in canned food spoilage. Curr Microbiol 61: 525–533. 20397018 10.1007/s00284-010-9648-8

[13] Loshon CA, Fliss ER, Setlow B, Foerster HF, Setlow P (1986) Cloning and sequencing of genes for small, acid-soluble spore proteins of *Bacillus cereus*, *Bacillus stearothermophilus* and “*Thermoactinomyces thalpophilus*”. J Bacteriol 167: 168–173. 3087949 10.1128/jb.167.1.168-173.1986 PMC212856

[14] Setlow B, Setlow P (1996) Role of DNA repair in *Bacillus subtilis* spore resistance. J Bacteriol 178: 3486–3495. 8655545 10.1128/jb.178.12.3486-3495.1996 PMC178117

[15] Clements MO, Moir A (1998) Role of the *gerI* operon of *Bacillus cereus* 569 in the response of spores to germinants. J Bacteriol 180: 6729–6735. 9852021 10.1128/jb.180.24.6729-6735.1998 PMC107780

[16] Paidhungat M, Setlow B, Driks A, Setlow P (2000) Characterization of spores of *Bacillus subtilis* which lack dipicolinic acid. J Bacteriol 182: 5505–5512. 10986255 10.1128/jb.182.19.5505-5512.2000 PMC110995

[17] Huang SS, Chen D, Pelczar PL, Vepachedu VR, Setlow P, et al (2007) Levels of Ca^2+^-dipicolinic acid in individual *Bacillus* spores determined using microfluidic Raman tweezers. J Bacteriol 189: 4681–4687. 17468248 10.1128/JB.00282-07 PMC1913426

[18] Zhang P, Kong L, Wang G, Setlow P, Li YQ (2010) Combination of Raman tweezers and quantitative differential interference contrast microscopy for measurement of dynamics and heterogeneity during the germination of individual bacterial spores. J Biomed Opt 15: 056010. 21054104 10.1117/1.3494567

[19] Kong L, Zhang P, Wang G, Setlow P, Li YQ (2011) Characterization of bacterial spore germination using integrated phase contrast microscopy, Raman spectroscopy and optical tweezers. Nat Protocols 6: 625–639. 21527920 10.1038/nprot.2011.307

[20] Bagyan I, Noback M, Bron S, Paidhungat M, Setlow P (1998) Characterization of *yhcN*, a new forespore-specific gene of *Bacillus subtilis* . Gene 212: 179–188. 9611260 10.1016/s0378-1119(98)00172-3

[21] Chen D, Huang SS, Li YQ (2006) Real-time detection of kinetic germination and heterogeneity of single *Bacillus* spores by laser tweezers Raman spectroscopy. Anal Chem 78: 6936–6941. 17007517 10.1021/ac061090e

[22] Williams RW, Cutrera T, Dunker AK, Peticolas WL (1980) The estimation of protein secondary structure by laser Raman. Spectroscopy from the amide III’ intensity distribution. FEBS Lett 115: 306–308. 6249643 10.1016/0014-5793(80)81193-8

[23] Kitagawa T, Hirota S (2002) Raman spectroscopy of proteins. In: Chalmers JM Griffiths PR, editors. Handbook of vibrational spectroscopy, vol. 5. Hoboken, NJ: John Wiley. 3426–3446.

[24] Zhang P, Kong L, Setlow P, Li YQ (2010) Characterization of wet-heat inactivation of single spores of *Bacillus* species by dual-trap Raman spectroscopy and elastic light scattering. Appl Environ Microbiol 76: 1796–1805. 20097820 10.1128/AEM.02851-09 PMC2837993

[25] Coleman WH, Chen D, Li YQ, Cowan AE, Setlow P (2007) How moist heat kills spores of *Bacillus subtilis* . J Bacteriol 189: 8458–8466. 17890306 10.1128/JB.01242-07 PMC2168948

[26] Coleman WH, Zhang P, Li YQ, Setlow P (2010) Mechanism of killing of spores of *Bacillus cereus* and *Bacillus megaterium* by wet heat. Lett Appl Microbiol 50: 507–514. 20302598 10.1111/j.1472-765X.2010.02827.x

[27] Benevides JM, Tsuboi M, Bamford JK, Thomas GJ Jr (1997) Polarized Raman spectroscopy of double-stranded RNA from bacteriophage phi6: local Raman tensors of base and backbone vibrations. Biophys J 72: 2748–2762. 9168049 10.1016/S0006-3495(97)78917-3 PMC1184471

[28] Benevides JM, Thomas GJ Jr (1983) Characterization of DNA structures by Raman spectroscopy: high-salt and low-salt forms of double helical poly(dG-dC) in H2O and D2O solutions and application to B, Z and A-DNA. Nucleic Acids Res 11: 5747–5761. 6889135 10.1093/nar/11.16.5747 PMC326311

[29] Foerster HF (1983) Activation and germination characteristics observed in endospores of thermophilic strains of *Bacillus* . Arch Microbiol 134: 175–181. 6615124 10.1007/BF00407754

[30] Foerster HF (1985) The effects of alterations in the suspending medium on low-temperature activation of spores of *Bacillus stearothermophilus* Ngb101. Arch Microbiol 142: 185–189.

[31] Setlow B, Cowan AE, Setlow P (2003) Germination of spores of *Bacillus subtilis* with dodecylamine. J Appl Microbiol 95: 637–648. 12911713 10.1046/j.1365-2672.2003.02015.x

[32] Kong L, Zhang P, Setlow P, Li YQ (2010) Characterization of bacterial spore germination using integrated phase contrast microscopy, Raman spectroscopy and optical tweezers. Anal Chem 82: 3840–3847. 20369827 10.1021/ac1003322

[33] Setlow P, Liu J, Faeder JR (2012) Heterogeneity in bacterial spore population. In: E Abel-Santos, editor. Bacterial spores: current research and applications. Norwich, UK: Horizon Scientific Press. 201–216.

[34] Setlow P, Johnson EA (2012) Spores and their significance. In: Doyle MP, Buchanan R, editors. Food microbiology, fundamentals and frontiers. Washington, DC: ASM Press. 45–79.

[35] Zhang P, Setlow P, Li YQ (2009) Characterization of single heat-activated *Bacillus* spores using laser tweezers Raman spectroscopy. Opt Expr 17: 16481–16491. 10.1364/OE.17.016480 19770863

[36] Ando Y (1980) Mechanism of nitrite-induced germination of *Clostridium perfringens* spores. J Appl Microbiol 49: 527–535. 10.1111/j.1365-2672.1980.tb04727.x 6260726

[37] Paidhungat M, Ragkousi K, Setlow P (2001) Genetic requirements for induction of germination of spores of *Bacillus subtilis* by Ca^2+^-dipicolinate. J Bacteriol 183: 4886–4893. 11466292 10.1128/JB.183.16.4886-4893.2001 PMC99543

[38] Peng L, Chen D, Setlow P, Li YQ (2009) Elastic and inelastic light scattering from single bacterial spores in an optical trap allows monitoring of spore germination dynamics. Anal Chem 81: 4035–4042. 19374431 10.1021/ac900250x PMC2717560

[39] Setlow B, Peng L, Loshon CA, Li YQ, Christie G, et al (2009) Characterization of the germination of *Bacillus megaterium* spores lacking enzymes that degrade the spore cortex. J Appl Microbiol 107: 318–328. 19302310 10.1111/j.1365-2672.2009.04210.x

[40] Heffron JD, Lambert EA, Sherry N, Popham DL (2010) Contributions of four cortex lytic enzymes to germination of *Bacillus anthracis* spores. J Bacteriol 192: 763–770. 19966006 10.1128/JB.01380-09 PMC2812458

[41] Vepachedu VR, Setlow P (2007) Role of SpoVA proteins in the release of dipicolinic acid during germination of *Bacillus subtilis* spores triggered by dodecylamine or lysozyme. J Bacteriol 189: 1565–1572. 17158659 10.1128/JB.01613-06 PMC1855772

